# Role of M1-polarized tumor-associated macrophages in the prognosis of advanced ovarian cancer patients

**DOI:** 10.1038/s41598-020-63276-1

**Published:** 2020-04-08

**Authors:** Antonio Macciò, Giulia Gramignano, Maria Cristina Cherchi, Luciana Tanca, Luca Melis, Clelia Madeddu

**Affiliations:** 1 Department of Gynecologic Oncology, Azienda Ospedaliera Brotzu, Cagliari, Italy; 2 Medical Oncology Unit, “Nostra Signora di Bonaria” Hospital, San Gavino, Italy; 3 Department of Medical Oncology, Azienda Ospedaliera Brotzu, Cagliari, Italy; 4 Department of Nuclear Medicine, Azienda Ospedaliera Brotzu, Cagliari, Italy; 5 Department of Medical Sciences and Public Health, University of Cagliari, Cagliari, Italy

**Keywords:** Cancer microenvironment, Ovarian cancer

## Abstract

The identification of prognostic and predictive markers is crucial for choosing the most appropriate management method for ovarian cancer patients. We aimed to assess the prognostic role of tumor-associated macrophage (TAM) polarization in advanced ovarian cancer patients. We carried out a prospective observational study that included 140 consecutive patients with advanced-stage high-grade serous ovarian cancer as well as patients with other histotypes of ovarian cancer and patients with ovarian metastasis from other sites between June 2013 and December 2018. Patients were enrolled at the time of laparoscopic surgery before receiving any antineoplastic treatment. We found that patients with high-grade serous papillary ovarian cancers had a prevalence of M1 TAMs, a higher M1/M2 ratio, and a longer overall survival (OS) and progression-free survival (PFS) than other patients. Regression analysis confirmed that there was a significant positive association between the M1/M2 ratio and an improved OS, PFS and platinum-free interval (PFI), both in the entire population and in patients stratified according to tumor type and initial surgery. Kaplan-Meier analysis was performed after the patients were divided into 2 groups according to the median M1/M2 ratio and revealed that patients with a high M1/M2 ratio had a higher OS, PFS and PFI than those with a low M1/M2 ratio. In conclusion, the prognostic and predictive role of TAM polarization in the tumor microenvironment could be of great clinical relevance and may allow the early identification of patients who are likely to respond to therapy. Further studies in a larger prospective sample are warranted.

## Introduction

Last year, Thorsson *et al*.^1^ examined over 10000 tumors comprising 33 cancer types and identified six immune phenotypes that define the tumor-associated immune status. Briefly, these clusters included 1) C1 (also called wound healing), which is associated with the high expression of angiogenic genes, high proliferation rates, and Th2 cells; 2) C2 (Interferon (IFN)-γ dominant)), which is associated with the highest level of M1/M2 macrophage polarization, high CD8 levels, and high T-cell receptor (TCR) diversity; 3) C3 (inflammatory), which is defined by the high expression of Th17 and Th1 genes and low levels of aneuploidy and copy number variation; 4) C4 (lymphocyte depleted), which is characterized by a high M2 response and Th1 suppression; 5) C5 (immunologically quiet), which shows the lowest lymphocytic responses and the highest M2 macrophagic responses; and 6) C6 (Tumor growth factor (TGF) β dominant), which is associated with the highest TGFβ expression and high lymphocyte infiltration (mixed type I and type II cells). For each subtype, the prevalence of the immune phenotype in specific neoplasms was described. In particular, ovarian cancer, cancer with highly BRCA mutation levels, gastric cancer, and cervical tumors were associated with the C2 signature. Thorsson *et al*.^1^ also demonstrated that each subtype was correlated with different prognoses in terms of overall survival (OS) and progression-free interval. In particular, when incorporating all prognostic features (immune subtypes, lymphocyte signatures, and Th17 vs Th1 vs Th2 cells) into Cox models fit for tumor type, stage, and both, the authors found that the prevalence of M1 macrophages, lymphocyte expression, a high number of TCR clonotypes, and cytokines released by activated Th1 and Th17 cells were associated with improved OS. In contrast, the wound healing (C1) signature, macrophage regulation (M2 prevalence), and TGFβ were associated with worse OS. Notably, ovarian cancer in the C2 cluster, including tumors with the highest M1 expression, was associated with the best prognosis in terms of OS.

Recently, when defining the role of macrophage polarization in the epithelial serous ovarian cancer microenvironment, we also found a prevalence of M1 cells with a high M1/M2 ratio among tumor-associated macrophages (TAMs)^2^. Further, this M1 polarization was associated with cancer-related anemia and was correlated with the severity of anemia, IL-6 levels, and iron metabolism changes, which, as we have already demonstrated, are typical of advanced stage ovarian cancer patients^3^. In light of Thorsson’s findings, we assessed the correlation of the M1 TAM percentage and M1/M2 ratio in the tumor microenvironment with patient prognosis. We used our previous cohort of high-grade serous ovarian cancer patients^2^ while increasing the sample size, and extending our evaluation to a cohort of patients with primary ovarian cancer with other histotypes (that are typically associated with worse prognosis and chemoresistance to platinum and its derivatives) and patients with ovarian metastasis from other sites.

## Results

From June 2013 to December 2018, we analyzed 140 consecutive patients with ascites secondary to primary ovarian cancer (stage IIIC-IV) or ovarian metastases from other sites, which included 95 patients with high-grade serous ovarian cancer and 45 additional patients with different histotypes of primary ovarian cancer (clear-cell, mucinous, and endometrioid) and ovarian metastases from other sites. Regarding surgery, 70% underwent initial cytoreductive surgery with radical intent (optimal cytoreductive surgery). Among these patients, 25% underwent one or more intestinal resections, 5% underwent splenectomy, 70% underwent parietal or total peritonectomy (including diaphragmatic peritonectomy). The remaining patients (30%) were not radically/optimally resectable and underwent diagnostic laparoscopy with minimal surgery (isolated omentectomy, adnexectomy, or peritoneal biopsy) to obtain a histological diagnosis. After surgery, all primary serous ovarian carcinoma patients received cisplatin-based chemotherapy according to standard guidelines (i.e., cisplatin/carboplatin + Taxol + bevacizumab).

**Figure 1:**
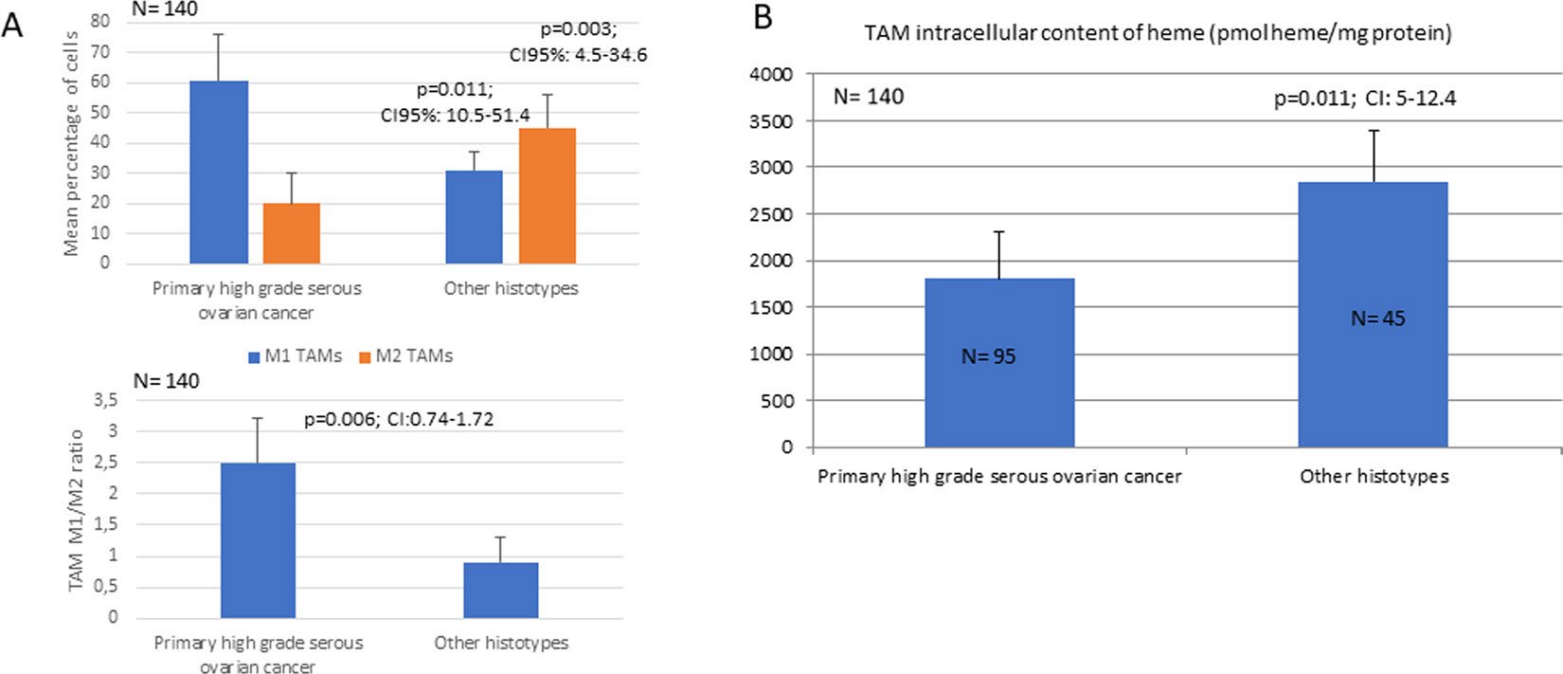
Analysis of tumor-associated macrophage (TAM) polarization. (**A**) M1 and M2 percentages and the M1/M2 ratio among TAMs isolated from primary high-grade serous ovarian cancer, different ovarian cancer histotypes and ovarian metastases from other sites. (**B**) Intracellular content of heme in TAMs isolated from patients with primary high-grade serous ovarian cancer, TAMs isolated from patients with different ovarian cancer histotypes, and patients with ovarian metastases from other sites.

**Figure 2:**
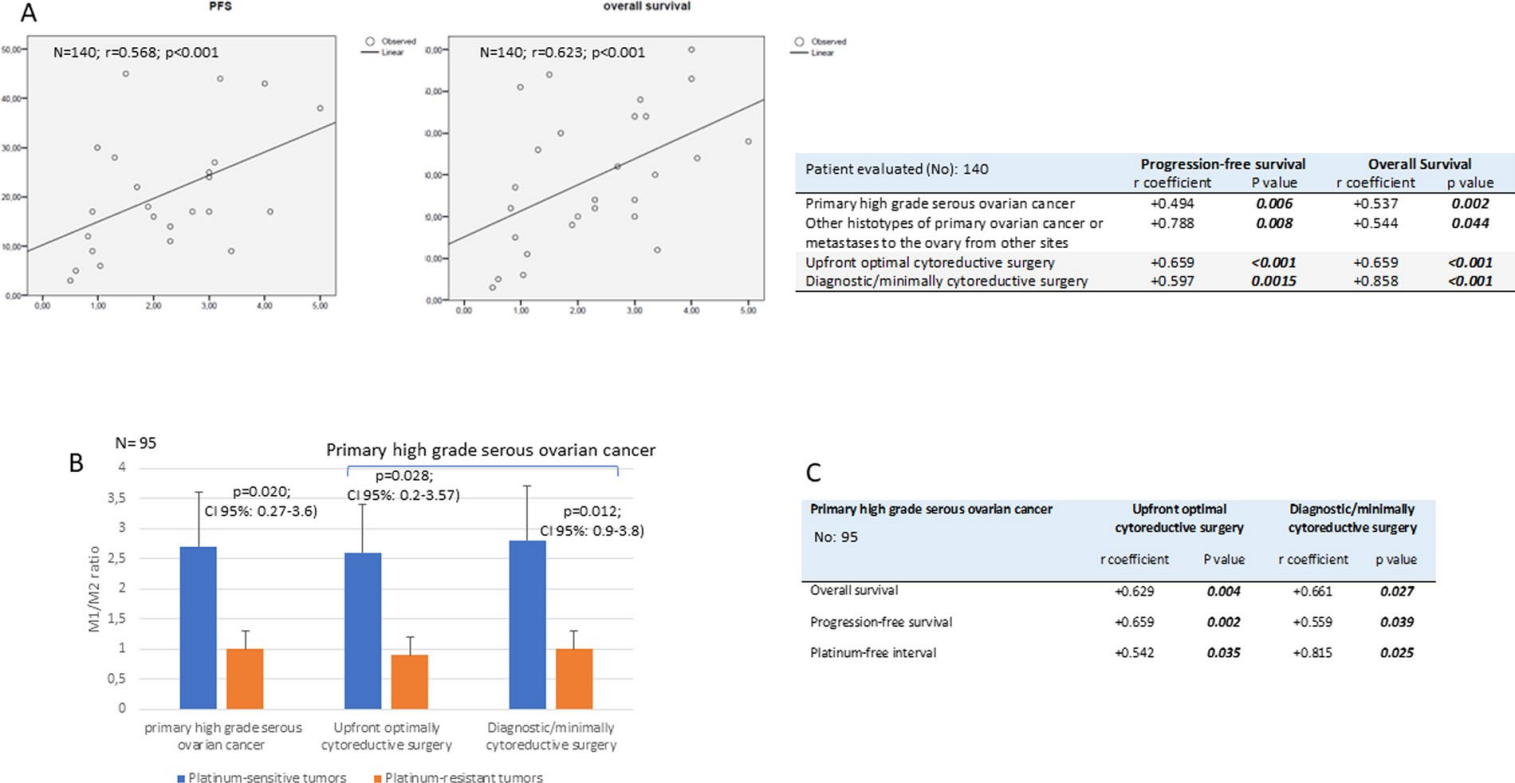
Correlation of tumor-associated macrophage (TAM) polarization with patient prognosis. (**A**) Regression analysis of the M1/M2 ratio and progression-free survival and overall survival in the entire cohort of patients and in patient subgroups based on tumor histotypes and the extent of surgery. (**B**) The M1/M2 ratio among TAMS isolated from primary high-grade serous ovarian cancer patients stratified based on platinum sensitivity. (**C**) Correlation analysis between the M1/M2 ratio and progression-free survival, overall survival and platinum sensitivity in patients with primary high-grade serous ovarian cancer stratified based on the extent of surgery.

In this cohort of patients, we found that TAMs isolated from the ascites of primary high-grade serous ovarian cancer patients presented a prevalence of M1 macrophages (CD14+/CD80+/Glut1+ cells) with a higher M1/M2 ratio than patients with different histotypes or ovarian metastasis from other sites (2.6 ± 0.8 vs 0.99 ± 0.4, 95% CI: 0.74–1.72; p = 0.006). In the latter group (data not yet published), the percentage of M1 cells (CD14+/CD80+/Glut+) was significantly lower (31 ± 10% vs 62.5 ± 18, 95% CI: 10.5–51.4; p = 0.011), and the percentage of M2 cells (CD14+/CD163+) was significantly higher (45 ± 11% vs 24 ± 6.9, 95% CI: 4.5–34.6; p = 0.003) than in the high-grade serous ovarian carcinoma group (Fig. 1A). Since M1 polarization is closely related to increased glycolytic activity, glucose uptake analysis showed a higher mean fluorescence intensity in TAMs isolated from primary high-grade serous ovarian cancer patients than in those isolated from patients with different ovarian cancer histotypes or ovarian metastasis from other sites. Moreover, since the unique energy metabolism of polarized cells as well as altered iron metabolism may influence the heme synthesis pathways, we assessed the TAM intracellular content of PpIX and heme. The assessment of intracellular PpIX showed significantly lower levels (% of positive cells, i.e., 8%±2 vs 60%±10, respectively) and fluorescence intensity signals (mean channel fluorescence, i.e., 20 AU vs 10 AU, respectively) in M1 macrophages (CD14+/CD80+) than in M2 macrophages (CD14+/CD163+). This was associated with a lower heme content in TAMs isolated from patients with primary high-grade serous ovarian cancer than in TAMs isolated from patients with different ovarian cancer histotypes and ovarian metastases from other sites (Fig. 1B). Thus, in M1 polarized cells, which are characterized by typical iron-sequestrating behavior, iron accumulated intracellularly and could not be incorporated into PpIX for heme synthesis due to defective glucose metabolism (due to the halted the Krebs cycle and lack of aminolevulinic acid synthesis).Regarding the analysis of how M1 polarization was associated with clinical prognostic parameters, notably, we found a positive relationship in the entire population between the M1/M2 ratio and OS (β coefficient = 0.623, 95% CI: 4.732–10.808; p < 0.001) and PFS (β coefficient = 0.568, 95% CI: 3.592–9.637; p < 0.001). This correlation was found both in patients with high-grade serous ovarian cancer (OS: β coefficient = 0.537, 95% CI 2.790–11.447, p = 0.002; PFS: β coefficient = 0.494, 95% CI 1.841–10.250, p = 0.006 for PFS) and patients with other histotypes or ovarian metastases from other sites (OS: β coefficient = 0.544, 95% CI 1.171–11.613, p = 0.022; PFS: β coefficient = 0.388, 95% CI 0.159–9.649, p = 0.044). When also evaluating the extent of surgery and analyzing separately patients who underwent initial optimal cytoreductive surgery (OS: β coefficient = 0.659, 95% CI 4.159–11–532, p < 0.001; PFS: β coefficient = 0.647, 95% CI 3.456–10.532, p < 0.001 for PFS) vs. diagnostic/minimal cytoreductive surgery (OS: β coefficient = 0.597, 95% CI 1.771–13.606, p = 0.015; PFS: β coefficient = 0.493, 95% CI 0.497–8.927, p = 0.039), the positive association between the M1 percentage, PFS, and OS remained (Fig. 2).

Similarly, by stratifying patients by tumor type, we found that patients with primary high-grade serous ovarian cancer had a significantly higher OS (28.3 ± 16.7 vs 15.4 ± 5.5 months, 95% CI: 3.63–22.08, p = 0.007) and PFS (21.1 ± 14.2 vs 10.1 ± 6.2 months, 95% CI: 3.01–18.99, p = 0.008) than patients with other histotypes and ovarian metastases from other sites.

Moreover, by analyzing separately patients with primary high-grade serous ovarian cancer, we found, as already reported in the literature^4^, that OS and PFS were not significantly different among patients who underwent optimal cytoreductive surgery compared to patients who underwent diagnostic/minimally cytoreductive surgery [median OS: 28.3 (range 3–60) vs 27.6 (range 5–60) months (95% CI −11.81–13.28; p = 0.514); median PFS 18.5 (range 3–60) vs 16 (range 5–45) months (95% CI −4.75–16.84; p = 0.786), respectively], yet OS and PFS was correlated with the M1 percentage and ratio in both groups (Fig. 2). Further, patient prognosis was not exclusively related to the extent of surgery (upfront optimal cytoreduction versus diagnostic/minimal cytoreduction) but seemed to be primarily dependent on the M1/M2 ratio, confirming the findings of Thorsson *et al*.^1^. Additionally, among high-grade serous primary ovarian cancer patients, those with platinum-sensitive tumors showed a significantly higher M1/M2 ratio than those with platinum-resistant tumors (2.6 ± 1.1 vs 0.7 ± 0.2, 95% CI 0.27–3.6; p = 0.020). This association was independent of the type of initial surgery. In fact, in both optimally debulked and diagnostic/minimally cytoreduced patients, platinum-sensitive tumors had a significantly higher M1/M2 ratio (optimally debulked: 2.7 ± 1.2 vs 0.7 ± 0.3, 95% CI 0.2–3.57; p = 0.028; diagnostic/minimally cytoreduced: 2.78 ± 0.94, p = 0.012) and higher OS (optimally debulked: 30.8 ± 15.49 months vs 4.7 ± 1.5 months, 95% CI 17.68–43.94, p = 0.001; diagnostic/minimally cytoreduced: 35.1 ± 16.2 months vs 8 ± 2.8 months, 95% CI 11.1–51.6, p = 0.005) and PFS (optimally debulked: 24 ± 13.9 months vs 4.67 ± 1.5 months, 95% CI 11.7–26.93; p = 0.031; diagnostic/minimally cytoreduced: 20.7 ± 10.5 months vs 4.5 ± 0.7 months, 95% CI 5.4 ± 30.2; p = 0.011) relative to patients with platinum-resistant cancers. Accordingly, a significant positive linear relationship (β coefficient = 0.504, 95% CI 0.493–10.334, p = 0.033) was found between the platinum-free interval and M1/M2 ratio (Fig. 2).

**Figure 3:**
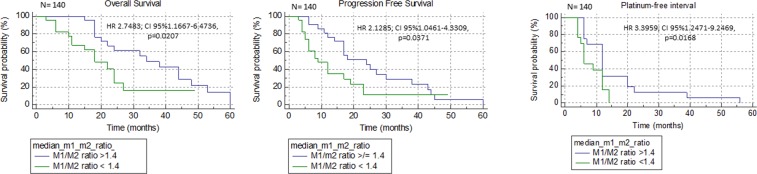
Kaplan-Meier survival curves comparing patients with high and low M1/M2 ratios. Patients with an M1/M2 ratio ≥ 1.4 showed a significantly higher overall survival (**A**), progression-free survival (**B**) and platinum-free interval (**C**) than patients with an M1/M2 ratio < 1.4.

The median value for the M1/M2 ratio was 1.4. Then, we analyzed the difference in OS and PFS by Kaplan-Meier curve analysis and log-rank analysis between patients with an M1/M2 ratio ≥ 1.4 and those with an M1/M2 ratio < 1.4. We found that patients with an M1/M2 ratio ≥ 1.4 had a significantly longer OS (34 months versus 18 months, HR 2.7483, 95% CI: 1.1667–6.4736; p = 0.0207), PFS (24 months versus 9 months, HR 2.1285, 95% CI: 1.0461–4.3309; p = 0.0371) and PFI (12 months versus 6 months, HR 3.3959, 95% CI 1.2471–9.2469; p = 0.0168) (Fig. 3).

## Discussion

The identification of predictive markers for chemotherapy sensitivity or resistance is crucial for choosing the most appropriate management strategy for ovarian cancer patients. In fact, maximal cytoreduction seems to offer the highest advantage in terms of PFS and OS only in chemoresistant tumors^4^. In this regard, emerging evidence from Thorsson *et al*.^1^ that found that M1 macrophage polarization, in the context of a peculiar immunological phenotype found in ovarian cancer patients, was associated with improved survival, was of great clinical relevance. Previously, in a heterogeneous population of ovarian cancer patients, Zhang *et al*.^5^ found that the overall M1/M2 ratio was inversely related to cancer stage and was positively related to overall survival, although they did not find a significant association in the different histotypes. Our present findings regarding the potential prognostic and predictive role of M1 macrophage polarization in the tumor microenvironment seem to confirm and further clarify these data. In fact, in this paper, serous papillary ovarian cancer that was more responsive to chemotherapy, and associated with longer OS, PFS and PFIs, had the highest percentage of M1 cells and the highest M1/M2 ratio in peritoneal effusions. In contrast, ovarian cancers typically known to be associated with chemoresistance and poor response to chemotherapy, and thus worse prognosis, such as mucinous and clear cell cancer and ovarian metastasis from other sites, had the highest percentage of M2 TAMs and a lower M1/M2 ratio. Therefore, we believe that our data, together with those from some previous papers^1,5^ may thus provide new insights for the early identification of patients who are likely to respond to treatment.

Other studies in the literature mostly assessed the prognostic role of M1 TAMs in preclinical ovarian cancer models and obtained heterogeneous and controversial results. For example, in a preclinical “*in vitro*” model, Cho *et al*.^6^ found that the treatment of cultured ovarian cancer cells with M1 macrophage conditioned media increased the metastatic potential of the cancer cells (migration and invasion activity) through the promotion of NF-kB activation. In contrast, Yin *et al*.^7^ reported that M2 TAMs were correlated with ovarian cancer progression and promoted spheroid formation during the early transcoelomic metastasis of ovarian cancer. It should be emphasized that differently from these works, we assessed macrophage polarization and the ratio “*ex vivo*” and not “*in vitro*”, thus reflecting the actual role of the ovarian cancer microenvironment in patient prognosis.

When examining why the highest levels of M1 TAMs were associated with the best prognosis of ovarian cancer patients who underwent conventional protocols that included surgery and chemotherapy, we believe it is necessary to hypothesize that there is a correlation between a greater efficacy of antineoplastic regimens and TAM M1 polarization.

It is known that during interactions with tumor cells, TAMs modify their activation state and polarization dynamically. Notably, the dynamic process of the phagocytosis of dying tumor cells by macrophages also affects their metabolic reprogramming, which turns out to be equivalent to the process that typically occurs in neoplastic cells (the Warburg effect)^9^. Indeed, Warburg *et al*.^9^ described this metabolic change in the tumor-associated macrophages, suggesting that this metabolic status can occur in cells other than cancer cells. The Warburg effect, which results in a shift towards anaerobic glycolysis and a reduction in oxidative phosphorylation, is also responsible for the lower values of intracellular PpIX and therefore of heme, which was well demonstrated in M1 TAMs from cancer ascites in the present work.

Therefore, it is reasonable to hypothesize that these unique glucose metabolism characteristics of neoplastic cells, which could be induced by the same mediators that lead immune cells to become M1 TAMs, as well as the associated M1-induced functional iron deficiency^2^, might cause M1-enriched tumors to be more sensitive to chemotherapy regimens. As we stated in our review on the mechanism of action of cisplatin^10^ and re-emphasized in our recent analysis (commentary) on the efficacy of poly(ADP‐ribose) polymerase inhibitors^11^, cancer cell metabolism based on an efficient Warburg effect is fundamental for the effectiveness of the antineoplastic actions of these drugs. It is worth noting here that in cancers that do not display the Warburg effect, such as in positron emission tomography-negative tumors, cisplatin is ineffective^10^. Consistently, increased levels of oxidative phosphorylation (OXPHOS) and higher intracellular levels of heme in cancer cells were shown to be associated with chemoresistance and thus with worse prognosis^12^. Additionally, a very recent, interesting paper highlighted the role of low heme levels in the promotion of apoptosis in cancer cells via the disruption of the electron transport chain^13^.

The well-known differences in iron metabolism that are typically associated with macrophage M1 polarization, which are described in detail in our recent work^2^ on the role of M1 TAMs in the tumor microenvironment in patients with advanced ovarian cancer, could also contribute to the (deficiencies in heme formation reported here. In fact, it is known that M1 macrophages are involved in iron accumulation and removal from tumor cells as a central mechanism of their defensive actions against cancer^14^; this therefore implies that the heme-related metabolic behaviors in the tumor microenvironment are very unusual.

Currently, the altered iron metabolism with functional iron deficiency has been only strictly associated with the development of anemia in chronic inflammation^2^, and its biological significance in the evolution of cancer has not yet been defined. It is important to highlight how Warburg^15^, who described altered energy metabolism in tumors for the first time was also among the first to highlight the indispensability of iron for cell proliferation in the literature^16^. Indeed, iron may contribute to tumor initiation, and it is also a fundamental nutrient that favors cancer cell proliferation. Iron is thus also essential for maintaining the rapid growth rate of cancer cells that require iron-dependent enzymes for DNA synthesis^17^. Moreover, iron plays a pivotal role in the function of OXPHOS and the tricarboxylic acid cycle. Iron is incorporated in subunits of complex I, II, III and IV of the respiratory chain, which is responsible for the transport of electrons and thus for the maintenance of mitochondrial membrane potential and ATP production^8^. In recent years, the discovery of different proteins, i.e., siderophore binding proteins, involved in iron metabolism and its intracellular import, storage and export has allowed us to better clarify the mechanisms that regulate the complex relationship between iron and cancer^18^. Thus, the use of anti-transferrin receptor mAbs^19^ and iron chelators^20^ were shown to effectively inhibit cancer cell growth^18^. Moreover, several studies have shown that iron depletion, such as that associated with M1 iron sequestering polarization^2^, may interfere with metabolic pathways that are fundamental for the activity of cisplatin and platinum-derived drugs. In this regard, it has been shown that cells that are iron starved accumulate in the G1 phase^21^, which is the cell cycle phase during which cisplatin acts^10^. Additionally, a p53 inducible subunit responsive to iron chelation and able to induce cell cycle arrest in the case of intracellular iron depletion has been found^22^.

From a prognostic point of view, it must also be emphasized that the increase in M1 macrophages is evidence of the presence of IFNγ, which is a mediator of M1 polarization; as a result, IFNγ is responsible for inducing the antineoplastic T cell-mediated response^23^. Therefore, the presence of this macrophage cell population is associated with a more antineoplastic immunological profile. In fact, M1-like macrophages are thought to promote antitumor immunity by supporting the antitumor Th1 response and cytotoxic T lymphocyte recruitment to tumors^24^.

However, it must be recognized that the presence of Interleukin (IL)−6, which is associated with M1 TAM polarization^2^, would modify this situation because it is recognized that IL-6 has immunosuppressive actions (such as inflammation) in the tumor microenvironment. The fact that IL-6 exerts a bivalent action is one of the most controversial aspects of the role of the immune system in the tumor microenvironment of advanced ovarian cancer, as demonstrated in our previous papers^25,26^. This aspect should, however, be better studied because while the role of IL-6 in the modification of iron metabolism through hepcidin is well established, its role in immune system modulation is different in the various phases of cancer progression^27^. Indeed, on the one hand, the rapid production of IL-6 contributes to host defense(by acting both on innate and adaptative immune cells and by linking them), while on the other hand, its excessive synthesis (and its trans-signaling dysregulation) results in the disruption of the immune response and leads to chronic inflammation and immunosuppression^28^.

In summary, it is reasonable to assume that there are at least two conditions that may improve the prognosis of patients with ovarian cancer undergoing chemotherapy that are characterized by the prevalence of M1 TAMS in the tumor microenvironment: 1) a metabolic profile that increases the efficacy of the currently used chemotherapy regimens via oxidative phosphorylation and functional alterations of iron; and 2) an immunological status characterized by a greater antineoplastic efficiency, which in combination with chemotherapy, helps to control tumor growth. There is no doubt that specific studies are needed to verify the antineoplastic role of these immune system components^29^ and their potential synergy with the currently used chemotherapy protocols.

## Methods

We carried out an observational perspective case series study, which included consecutive patients (depending on the daily availability of our laboratory) with ascites secondary to primary ovarian cancer (stage IIIC-IV) or metastases to the ovary from other sites from June 2013 to December 2018. Patients were evaluated at the time of laparoscopic surgery before receiving any antineoplastic treatment. The study was approved by the Local Institutional Independent Ethics Committee of the “Azienda Ospedaliero Universitaria”, Cagliari, Italy and was carried out in accordance with the principles of the Declaration of Helsinki. All enrolled patients provided written informed consent for participation in the study and for the use of their biological samples for laboratory analyses.

### Measures and outcomes

Patient samples (ascitic fluid and peripheral blood) were collected from enrolled patients. TAMs from ascites were isolated, and their functional/metabolic phenotype and polarization were assessed according to previously reported procedures. TAM polarization was correlated with patient prognostic factors, i.e., OS, progression-free survival (PFS) and the platinum-free interval (PFI). Progression-free survival (PFS) and OS were defined as the time from randomization to objective disease progression based on imaging (according to modified Response Evaluation Criteria in Solid Tumors, RECIST, version 1.1) or death from any cause, respectively. The platinum-free interval (PFI) was defined as the time from the end of platinum-based chemotherapy to the diagnosis of relapse.

### TAM isolation

Immediately after collection, ascites was centrifuged at 300 × g for 10 min at room temperature to obtain a cell pellet. Supernatants were then frozen at −80 °C until the assay. Tumor-associated macrophages (TAMs) were isolated with a Ficoll-Hypaque (Fresenius Kabi, Morge AS, Oslo, Norway) double density gradient into two distinct layers (100% and 75% Ficoll-Hypaque) by centrifugation at 300 × g for 30 min at room temperature. TAMs formed a clear band on top of the 75% Ficoll-Hypaque. TAMs were collected, washed twice in Hanks balanced salt solution (GIBCO, Carlsbad, CA, USA), and counted. Their viability was tested by the Trypan blue dye exclusion test. TAMs were then kept in complete medium consisting of RPMI 1640 medium (Sigma, St. Louis, MO, USA) with 20% fetal calf serum (GIBCO) and 10 mg/mL gentamicin (GIBCO).

### Assessment of TAM polarization and functional status

Immediately after separation, the TAM phenotype was evaluated by a CD14 mAb. The assessment of TAMs by CD14 staining showed that CD14 + cells were the largest cell population among monocytes (86.8%), with less than 20% contamination by non-lympho-monocyte cells and more than 90% viable cells. Tumor-associated macrophages were also gated on a forward scatter versus 90-degree light scatter or side scatter plot. Data were acquired and analyzed by flow cytometry (FACScan, Beckton Dickinson, Franklin Lakes, NJ, USA) using CellQuest software (BD Biosciences, San Jose, CA, USA). To determine the M1 and M2 polarization phenotype, cells were stained with FITC-labeled CD14, anti-human PE-labeled CD80 (BD Biosciences) or anti-human PE-labeled CD163 (Miltenyi Biotec GmbH, Bergisch Gladbach, Germany), and anti-human-Glut-1 receptor (Abcam, Cambridge, MA, USA) or an anti-human HLA-DR (BD Biosciences, San Jose, CA, USA); anti-human APC-labeled CD206 (BD Biosciences, San Jose, CA, USA), or an intracellular anti-human Arginase-1 (RD System, Minneapolis, MN, USA) APC labeled monoclonal antibody (mAb). Cell suspensions of TAMs (100 μL) at a concentration of 1 × 10^6^ cells/mL were incubated with 5–10 μL of each mAb for 15 min at 4 °C, washed twice with PBS, and fixed with 1% paraformaldehyde. For intracellular staining, cells were washed, subjected to surface antigen staining and fixed as described above. Then, fixed cells were washed, permeabilized with a permeabilizing solution (BD Biosciences) according to the manufacturer’s instructions and stained with intracellular mAb in permeabilization buffer (BD Biosciences). Moreover, to confirm the functional activation and the metabolic profile that is typical of M1-polarized TAMs, glucose uptake was evaluated in CD14+/CD80 + and CD14+/CD163 + cells by using 2-[N-(7-nitrobenz-2-oxa-1,3-diazol-4-yl) amino]-2-deoxy-D-glucose (2-NBDG), a fluorescently labeled deoxyglucose analogue (Glucose Uptake Cell-Based Assay Kit, Cayman Chemical, Ann Arbor, MI, USA). In this assay, TAMs at a density of at least 5 × 10^5^ cells/mL were treated in 100 μL glucose-free medium containing 150 μg/mL 2-NBDG. Cells were incubated for 10 min, harvested in a plastic tube and centrifuged for 5 min at 400 × g at room temperature. The supernatant was aspirated, and 1 mL of assay buffer was added to each tube. This step was repeated twice, and cells were analyzed immediately by flow cytometry. Cells that take up 2-NBDG display fluorescence with excitation and emission at 485 nm and 535 nm, respectively. To assess the functional status of TAMs at the time of collection, cell cycle analysis was performed. Cells (1 × 10^6^/mL) were washed twice in RPMI 1640, fixed in ice-cold 70% ethanol, and stored at 4 °C. After washing, cells were suspended in PBS and 2 mL of propidium iodide (50 μg/mL; Sigma, St. Louis, MO, USA) plus 25 μL of RNAse (1 mg/mL; Sigma) and incubated for 20 min at room temperature. Cells were analyzed using a FACScan cytometer and Modfit software (BD Biosciences). The results were calculated as the percentage of cells and the mean fluorescence intensity (MFI).

Moreover, since both glucose (through the Krebs cycle) and iron metabolism have a significant influence on the heme synthesis pathways, we assessed the TAM intracellular content of heme and its precursor protoporphyrin IX (PpIX). The intracellular accumulation of PpIX was examined by flow cytometry, and all steps were carried out in dim light to avoid PpIX photobleaching^30^. The excitation light (argon laser 1.5 mW) was 488 nm, and the PpIX fluorescence from 10,000 cells was measured with a 630 nm bandpass filter (FL4 (PpIX), 630 ± 11 nm). In addition to PpIX fluorescence, the cells were simultaneously analzyed for the expression of CD14(FITC)/CD80(PE) and CD14(PE)/CD163(FITC)/CD122(PErCP) as markers of macrophage polarization using fluorescently labeled antibodies. Following staining, cells were washed, fixed in 2% formaldehyde and immediately analyzed using a modified flow cytometer (FACScan, Becton Dickinson) capable of four-color acquisition. Isotype controls were used to determine the levels of nonspecific binding. The analysis of heme was carried out using a commercially available heme assay kit (Sigma Aldrich, Merck KGaA, Darmstadt, Germany). This assay is based on an aqueous alkaline solution method, in which heme is converted into a uniform colored form, producing a colorimetric (400 nm) result that is directly proportional to the heme concentration in the sample. The optimized formulation reduces interference and exhibits high sensitivity. A concentration of 1 mg/dL heme equals 15.3 M, 0.001%, or 10 ppm. The kit has a linear detection range of 0.6–125 M heme in a 96-well format^31^.

### Statistical analysis

Based on a 90% probability of detecting a relationship between macrophage polarization (with the M1/M2 ratio as the independent variable) and the other dependent variables (OS and PFS) at a two-sided significance level of 0.01, we planned to enroll at least 100 patients in the original study. Data are reported as the mean ± SD. All tests were performed on all patients enrolled. Differences between means were examined by a 2-tailed, unpaired t test for normally distributed variables or by the Mann-Whitney test for non-normally distributed variables. Correlations were tested by Pearson correlation analysis (or Spearman, if necessary) using Bonferroni’s correction for multiple comparisons. Linear regression analysis was also used to test the relationship between the M1/M2 ratio (independent variable) and OS and PFS (dependent variables) and to confirm its predictive value. The M1/M2 ratio was divided into high and low levels based on the median value. Then, survival analyses were performed after the patients were divided into 2 groups according to the median M1/M2 ratio using Kaplan-Meier curves and log-rank analysis to compare OS, PFS and the PFI between groups. HRs and CIs were calculated from univariate Cox regression survival analysis. All reported p-values are 2-tailed, and p  <  0.05 was considered statistically significant.

## Data Availability

The datasets generated during and/or analysed during the current study are available from the corresponding author on reasonable request
